# Formation of Ultrathin and Highly Stable Aromatic Monolayers on Silver Surface—Three Legs Are Better Than One

**DOI:** 10.1021/acsami.5c19530

**Published:** 2026-03-03

**Authors:** Anna Rojek, Daria M. Cegiełka, Mateusz Wróbel, Magdalena Stępień, Yoshiaki Shoji, Takanori Fukushima, Michael Zharnikov, Piotr Cyganik

**Affiliations:** † 37799 Jagiellonian University, Faculty of Physics, Astronomy and Applied Computer Science, Smoluchowski Institute of Physics, Łojasiewicza 11, Kraków 30-348, Poland; ‡ Laboratory for Chemistry and Life Science, Institute of Integrated Research, 530262 Institute of Science Tokyo, Yokohama 226-8501, Japan; § Research Center for Autonomous Systems Materialogy (ASMat), Institute of Integrated Research, Institute of Science Tokyo, Yokohama 226-8501, Japan; ∥ Angewandte Physikalische Chemie, 9144 Universität Heidelberg, Im Neuenheimer Feld 253, Heidelberg D-69120, Germany

**Keywords:** self-assembled monolayers, triptycene, thermal stability, chemical stability, XPS, NEXAFS spectroscopy, IRRAS

## Abstract

Surface functionalization by self-assembled monolayers (SAMs) is a key method for controlling the morphology and electronic coupling of organic semiconductors (OSC) on metal electrodes in organic electronic and photovoltaic devices. For such applications, it is crucial to use ultrathin, and thus highly conducting, upright-oriented aromatic monolayers (compatible with OSC growth), which should also possess high thermal and chemical stability, to withstand both the OSC deposition procedure and heat dissipation during device operation. Following these criteria, we analyze here the thinnest possible aromatic SAMs on the most conductive metal electrode, silver. These SAMs are built from molecules with a thickness corresponding to a single phenyl ring and are designed using either traditional monodentate or triptycene-based, tridentate surface anchoring geometry. Spectroscopic analysis confirms, in both cases, the successful formation of well-defined, upright-oriented, and ultrathin (∼0.8 nm) aromatic monolayers, with all available anchoring groups bonded to the metal substrate, enabling direct comparison of both structural designs. Quantitative thermal and chemical stability analysis shows inferior characteristics for the standard monodentate anchoring design. In contrast, the application of triptycene-based tripods leads to the formation of ultrathin monolayers with high thermal and chemical stability, which is comparable to—or even greater than—that of significantly thicker monodentate SAMs. These monolayers thus meet the criteria for optimal interface engineering in organic electronics and photovoltaics, opening new opportunities for improved device performance.

## Graphical abstract

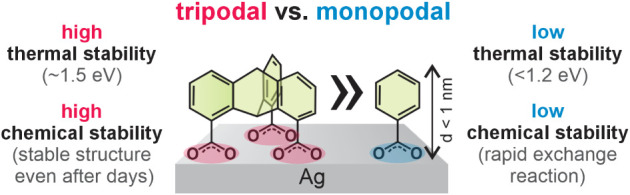

## 1 Introduction

Interfaces between the metal electrodes and an organic semiconductor (OSC) are a crucial part of organic electronic and photovoltaic devices. It is well established that the OSC growth and its electronic coupling with the metal electrodes can be significantly promoted by the formation of an aromatic self-assembled monolayer (SAM) chemically bound to the electrode by suitable anchoring groups and exposing a vertically oriented aromatic framework toward the OSC.^1–6^ The organic electronic and photovoltaic devices in which SAMs are often used for electrode functionalization are frequently exposed to demanding conditions, including thermal, chemical, and environmental stresses. This includes not only the fabrication process but also the regular operation of these devices. The fabrication process, e.g., thermal annealing of the active layer or electrode deposition, often involves temperatures in the range of up to ∼430 K.^7,8^ A similar range of thermal stability is also crucial for the performance of organic solar cells.^3,9,10^ Thermal stress can induce desorption, chemical decomposition, and interfacial reconstruction. This can happen especially when substrate–SAM bonding is weak. Additionally, exposure to ambient conditions, including oxygen, water, and light, can lead to undesirable degradation of the organic–inorganic interfaces.

Taking all these factors into account, the thermal and chemical robustness of SAMs is a key aspect for the design of organic electronic and photovoltaic devices.^10–13^ The properties of SAMs are, however, substrate-specific and should be adapted to a particular material serving as electrodes in these devices. One such material is silver, which is used much more frequently for this purpose compared to gold, especially in organic and perovskite solar cells. This is mainly due to its approximately 100 times lower price, the highest electrical conductivity among metals, and its application in the form of ink for printing flexible circuits.^14^ Significantly, functionalization of silver with SAMs is well established and can be used as a basis for the design of the relevant interfaces.^15–19^ Consequently, SAM-modified silver electrodes are frequently used in such devices as organic light-emitting diodes (OLEDs), organic thin-film field-effect transistors (OTFTs), etc., enhancing their performance.^12,20,21^ However, possible degradation of these devices under thermal or electrical stress remains a problem, limiting their operation. Therefore, enhancing the SAM stability on Ag surfaces is highly relevant, as it can help to preserve the energy level alignment and electrical conductance across the interfaces during device operation. This is particularly important for photovoltaic applications, where interfacial degradation is recognized as a major contributor to the performance loss over time.^12,13,22,23^

Taking into account the above considerations and further relevant aspects in the context of using SAMs for functionalization of metal electrodes in organic electronics and photovoltaics, it is desirable to (1) keep the thickness of the SAMs as small as possible to maximize the charge transport across the interfacial layer, (2) maximize the thermal and chemical stability of the SAM, which is necessary to survive both the OSC deposition procedure and the device operation, and (3) have SAMs with a structural design allowing the introduction of proper functional groups, which can promote favorable OSC morphology and lower the injection barrier to the OSC for the charge carriers by changing the work function of the electrodes. Following the above criteria for the SAM design, in the current study, we analyze the thinnest possible aromatic SAMs on the silver substrate built by molecules with a thickness corresponding to a single phenyl (Ph) ring, designed using two different concepts. The first concept, most common for the SAM formation, exploits PhCOO molecules, which contain a single phenyl unit and bind in a monopodal fashion to the silver surface by the carboxylic acid (CA) anchoring group (Figure 1).^18^ The alternative concept involves triptycene-based TripCOO molecules, representing a rigid propeller-shaped structure consisting of three phenyl rings decorated with CA groups capable of anchoring to the silver substrate in a tripodal fashion (Figure 1).^19,24^ Taking these two systems as representative examples, we address in the current study their thermal and chemical stability and its potential dependence on tripodal versus monopodal anchoring configurations to pursue an optimal design concept for interface engineering in organic electronics and photovoltaics.

**Figure 1:**
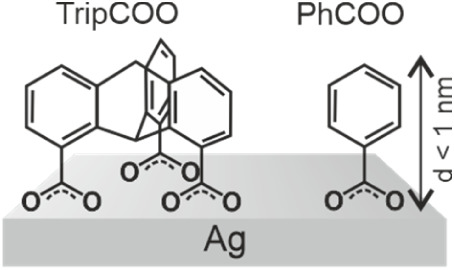
Structures of TripCOO and PhCOO adsorbed on Ag over the CA group(s) in the tripodal and monopodal bonding configurations, respectively.

The idea of multiple bonding as a possible way to increase the stability of ligands chemisorbed on metals is not new and originates from the *chelate effect,* ^25^ where multiple bonding provides higher thermodynamic stability driven by the disorder entropy change during bonding with metal, which is lower for multipodal ligands compared to monopodal ones. Along with the chelate effect, *bridging effects* can also be involved to some extent. It is, however, unclear to what extent these effects, with either individual COO anchors or the entire TripCOO adsorbate serving as a bridging ligand, are relevant in the given case. Importantly, in contrast to the formation of isolated metal complexes, for which the *chelate* and *bridging effects* are usually considered, the molecules forming SAMs are bonded to the metal surface.^26^ In such cases, by adding additional enthalpic and entropic contributions arising from the surface chemisorption process, such as two-dimensionally (2D) ordered structure formation by ligands, molecule-metal bonding reconfiguration, and metal surface relaxation/reconstruction, a theoretical prediction of the system stability becomes extremely challenging.^27^ The dedicated experiments are then in fact the only way to find out if the multipodal bonding concept is energetically favorable and, most importantly, whether it leads to the desired improvement in both thermal and chemical stability of a particular monolayer. We note at this point that for many SAMs these two types of stabilities are in fact mutually exclusive, i.e., an improved chemical stability might be achieved at the price of thermal stability reduction.^28–30^ Moreover, in many cases, the bonding of multipodal molecules to the metal substrate does not involve all potential anchoring groups, which is yet another factor that needs experimental verification.^26^ So far, several types of multipodal anchoring have been tried for SAMs, mainly based on thiols, including bipodal,^26,31–36^ tripodal^19,24,26,37–51^ or even tetrapodal^45,52,53^ configurations. However, to the best of our knowledge, there is just a handful of qualitative studies, in which an improved thermal stability of bipodal^33–36^ or tripodal^37^ configurations on gold substrate relative to monopodal anchoring was demonstrated. Moreover, in these studies, only SAMs with an aliphatic backbone and thiol anchoring groups were tried.

In this study, after the spectroscopic verification of the quality, parameters, and adsorption configuration of the TripCOO and PhCOO SAMs, we conducted quantitative thermal and chemical stability analysis of these monolayers. The thermal stability analysis included the desorption energy estimation, while the chemical stability analysis was based on the exchange process with potential molecular substituents capable of forming SAMs on the same substrate. Our results unequivocally show that the application of triptycene-based tripodal monolayers drastically improves the thermal and chemical stability of ultrathin aromatic SAMs compared to the monopodal systems based on phenyl, which have very low stability, rendering it hardly useful for any meaningful applications. Moreover, the ultrathin (∼0.8 nm) aromatic SAMs based on triptycene show significantly higher thermal stability (∼0.2 eV higher desorption energy) compared to the noticeably thicker monolayers of long-chain alkanethiols and are strongly resistant to being exchanged by these molecules in the respective solution.

## 2 Results and Discussion

### 2.1 Spectroscopic Characterization of TripCOO/Ag and PhCOO/Ag

Representative X-ray photoelectron spectroscopy (XPS) and near-edge X-ray absorption fine structure (NEXAFS) spectroscopy data for TripCOO/Ag and PhCOO/Ag are listed in Figure 2. The C 1s XP spectrum of TripCOO/Ag (Figure 2a, top spectrum) is dominated by an asymmetric peak at ∼283.5 eV, corresponding to the triptycene framework, in agreement with the former analysis.^19^ This peak can be tentatively decomposed into two components at binding energies (BEs) of ∼283.5 eV (1) and ∼284.3 eV (2). The less intense peak located at a higher BE of ∼287.0 eV (3) corresponds to the carbon atoms in the carboxylate (COO^–^) anchoring groups bound to the silver substrate.^15–19^ The symmetric bidentate character of this bonding for all three carboxylate groups of TripCOO is directly confirmed by the respective O 1s XP spectrum, which exhibits a single peak located at a BE of ∼530.1 eV (Figure 2b, top spectrum).^15–19^ Similarly, the C 1s XP spectrum of monopodal PhCOO/Ag (Figure 2a, bottom spectrum) exhibits the main peak at a BE of ∼283.9 eV (1) and an additional peak at ∼287.0 eV (2), corresponding to the phenyl backbone and the COO^–^ anchoring group, respectively.^18^ Also, similar to TripCOO/Ag, the O 1s XP spectrum of PhCOO/Ag shows a single peak at a BE of ∼530.1 eV (Figure 2b, bottom spectrum), corresponding to the formation of bidentate bonding with the silver substrate by the single anchoring carboxylate group (two binding oxygen atoms per molecule compared to six in TripCOO/Ag).

**Figure 2:**
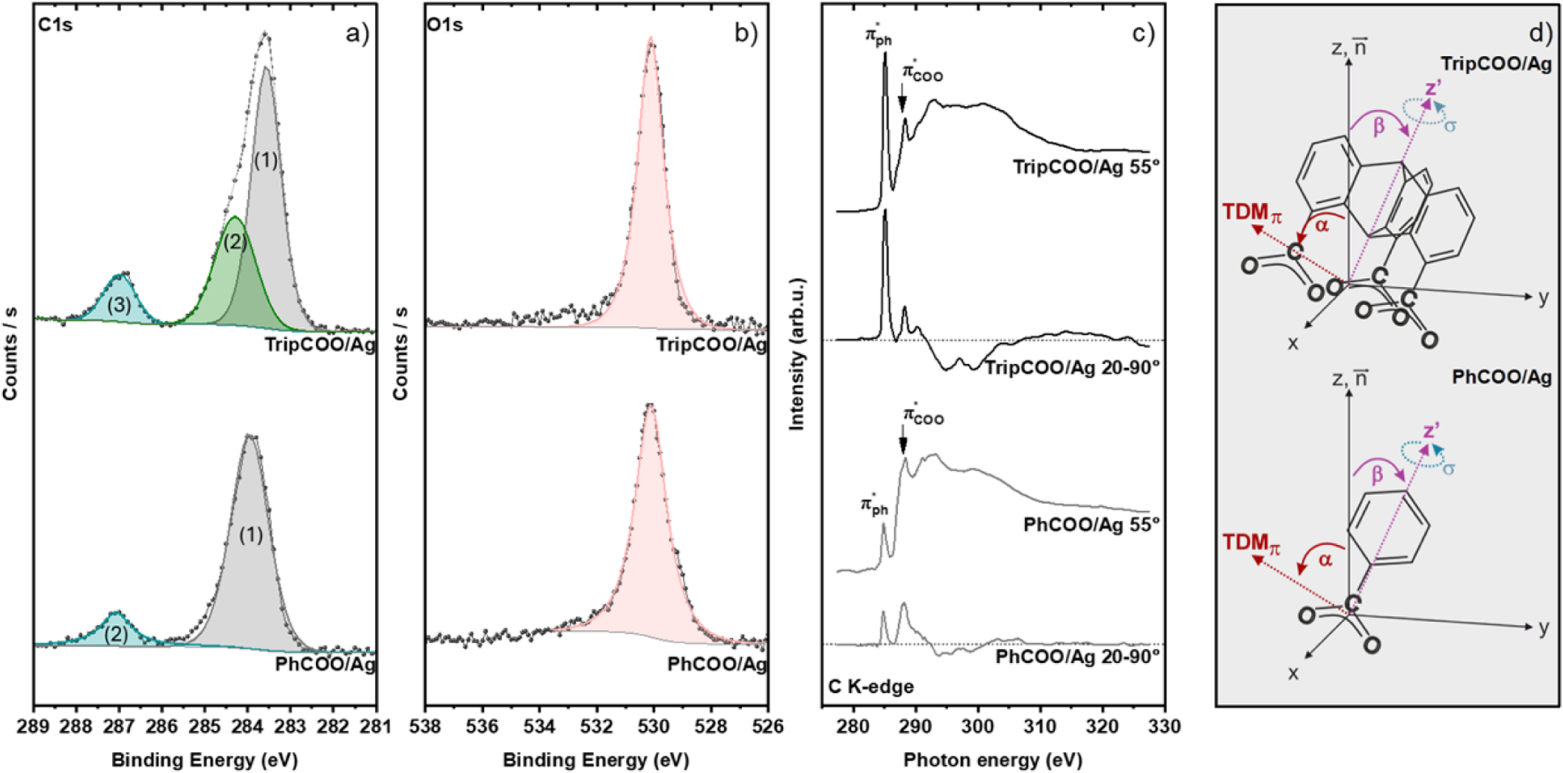
Representative XPS and NEXAFS data for TripCOO/Ag and PhCOO/Ag. (a) C 1s XP spectra of the SAMs decomposed into individual components marked by numbers. (b) O 1s XP spectra of the SAMs. (c) C K-edge NEXAFS spectra of the SAMs taken at an X-ray incidence angle of 55° (magic angle) and the difference between the spectra collected at the normal (90°) and grazing (20°) incidence, with the **E** vector being parallel and nearly perpendicular to the sample surface, respectively. (d) Schematic of the orientation of TripCOO and PhCOO in the SAMs relative to the surface normal (*z*-axis) established by the NEXAFS analysis. The angle defining the orientation of the transition dipole moment (TDM) of the π* orbitals (α) and the molecule tilt angle (β) are indicated. The TDM_π_ is perpendicular to the planes of the aromatic rings (for TripCOO/Ag, only one of the three TDMs is shown for the sake of clarity).

The XPS data were also used for the calculation of the effective SAM thicknesses using the standard method^54^ based on the C 1s/Ag 3d intensity ratios. We assumed exponential attenuation of the photoelectron signal with the literature values of the attenuation lengths^55^ and used the hexadecanethiol/Ag (CH_3_-(CH_2_)_16_-S/Ag, HDT/Ag) as a reference system^56^ with the known film thickness (see the Supporting Information for details). The obtained values of the effective thicknesses (summarized in Table 1) of 0.83 ± 0.05 and 0.69 ± 0.05 nm for TripCOO/Ag and PhCOO/Ag, respectively, are close to the geometrical thickness (∼0.88 nm) of these monolayers, assuming an upright molecular orientation (see Figure 1). Note that the XPS-derived thickness is not the geometric one but depends on the packing density and the concentration of defects. Therefore, we assume that the slightly lower (by ∼0.14 nm) thickness of the PhCOO SAM compared to the TripCOO monolayer reflects predominantly the somewhat lower structural quality of the former film, which is consistent with the former STM analysis.^18,19^

**Table 1:** Summary of the Parameters Derived for TripCOO/Ag, PhCOO/Ag, HDT/Ag, and HDCOO/Ag

	TripCOO/Ag	PhCOO/Ag	HDT/Ag	HDCOO/Ag
SAM thickness [Å]	8.3 (±0.4)	6.9 (±0.5)	20.4	14.9 (±1.5)
Tilt angle of SAM (φ) [°]	22 (±3)	24 (±3)^a^	—	—
Desorption temperature [K]	495 (±16)	385 (±46)	427 (±15)	403 (±308)
Desorption energy [eV]	1.51 (±0.05)	1.16 (±0.13)	1.29 (±0.04)	1.22 (±0.08)

a Assuming the twist angle of 32° for PhCOO/Ag.

Complementary information is provided by the C K-edge NEXAFS data summarized in Figure 2c. The electronic structure of TripCOO/Ag and PhCOO/Ag is best represented by the spectra acquired at the so-called magic incidence angle of the X-ray beam (∼55°), which are not affected by molecular orientation.^57^ For TripCOO/Ag, this spectrum exhibits two resonances characteristic of its chemical structure, i.e., a pronounced π_1_* resonance of the phenyl rings at ∼285.1 eV ( $\pi_{ph}^{*}$ in Figure 2c) and a further π* resonance at ∼288.2 eV associated with the carboxylate anchoring groups ( $\pi_{COO}^{*}$ in Figure 2c).^19^ The same two characteristic resonances are also visible for PhCOO/Ag, which is fully consistent with former NEXAFS spectroscopy studies of this monolayer.^18^ The intensities of these resonances are, however, noticeably lower than in the TripCOO/Ag case, which suggests a somewhat inferior quality of the PhCOO monolayer.

For both SAMs, pronounced linear dichroism was observed, as confirmed by the difference spectra between the data taken at normal (90°) and grazing (20°) incidence of the X-ray beam (Figure 2c). The positive peaks at the positions of the π* resonances in the difference spectra for both monolayers confirm an upright orientation of the phenyl moieties,^58^ consistent with the effective film thicknesses being close to the geometric ones, as obtained from XPS. To estimate the average molecular tilt angle β in TripCOO/Ag and PhCOO/Ag (see Figure 2d), the dependence of the $\pi_{ph}^{*}$ intensity on the X-ray incidence angle was analyzed (Figure S2 and Figure S3) within a theoretical framework for a vector-type orbital,^57^ following a former study (see the Supporting Information for more details).^18,19,57^

The calculated average molecular tilt angles (summarized in Table 1) are ∼22° and ∼24° for TripCOO/Ag and PhCOO/Ag, respectively. Whereas the value obtained for PhCOO/Ag is fully consistent with former studies of this system,^18^ the value obtained for TripCOO is by ∼10° higher in the present case, which we attribute to the different types of substrates used in both experiments, i.e., a 100-nm-thick solid Ag/Si substrate used in our study compared to the two-atomic-layer-thick underpotentially deposited Ag film on the atomically flat Au/mica support used previously.^19^ Even though the use of polycrystal (e.g., metal films on silicon as in our study) or monocrystal (e.g., epitaxially grown metal films on mica) metal substrates does not noticeably affect the molecular inclination for particular SAMs,^59,60^ the use of just a two-atomic-layer-thick Ag substrate instead of a solid one may change the involvement of the substrate adatoms in the bonding configuration of a given SAM. Note that such involvement is well documented for thiols and selenols on Ag(111)^29,61^ and also cannot be excluded for the carboxylic anchoring group. Consequently, some of the anchoring groups of TripCOO can be bonded to the Ag adatoms, while the other groups are bonded to the “in-plane” surface atoms, resulting in tilting of the entire triptycene framework.

Summarizing the results of the XPS and NEXAFS spectroscopy characterization of TripCOO/Ag and PhCOO/Ag, we conclude that, in both cases, well-defined monolayers are formed by the upright-oriented molecules, with all available anchoring carboxylate groups involved in chemical bonding with the substrate in the bidentate configuration. Consequently, these two ultrathin aromatic SAMs, formed on the application-relevant metal substrate, can be compared with regard to the impact of the monopodal versus tripodal bonding configuration on their thermal and chemical stability.

### 2.2 Thermal Stability Analysis

To quantitatively assess the thermal stability of TripCOO/Ag and PhCOO/Ag, temperature-programmed XPS (TP-XPS) analysis was applied. The C 1s, O 1s, and Ag 3d signal intensities were monitored as functions of temperature, which was continuously ramped from RT to 650 K at a rate of 3.6 K/min. The spectra were recorded every 5 K, each time at a new spot on the sample to avoid irradiation-induced damage.

For TripCOO/Ag, a sharp drop in the C 1s signal intensity associated with the triptycene backbone (peaks 1 and 2 in Figure 2a) was recorded (Figure 3a), following the progressive decrease in this intensity at the joint level of 10%. This decrease is most likely related to the desorption of some physisorbed advantageous carbon from the sample surface. In contrast, the sharp drop corresponds to the molecular desorption with a characteristic temperature of *T*_D_ ∼505 K, as determined from the minimum of the C 1s signal derivative (Figure 3b). The latter conclusion is supported by the behavior of the Ag 3d signal (Figure 3c), which shows a strong increase in intensity at *T*_D_ (indicated by the vertical dashed line) associated with reduced attenuation of the given signal by the partly desorbed monolayer. The behavior of the triptycene backbone signal is also well correlated to those of the C 1s (peak 3 in Figure 2a) and the O 1s (Figure 2b) signals associated with the carboxylate anchoring groups (Figure 3d). This correlation indicates the desorption of the entire molecules, most likely by breaking the bonds between the anchoring groups and the substrate (O–Ag).

**Figure 3:**
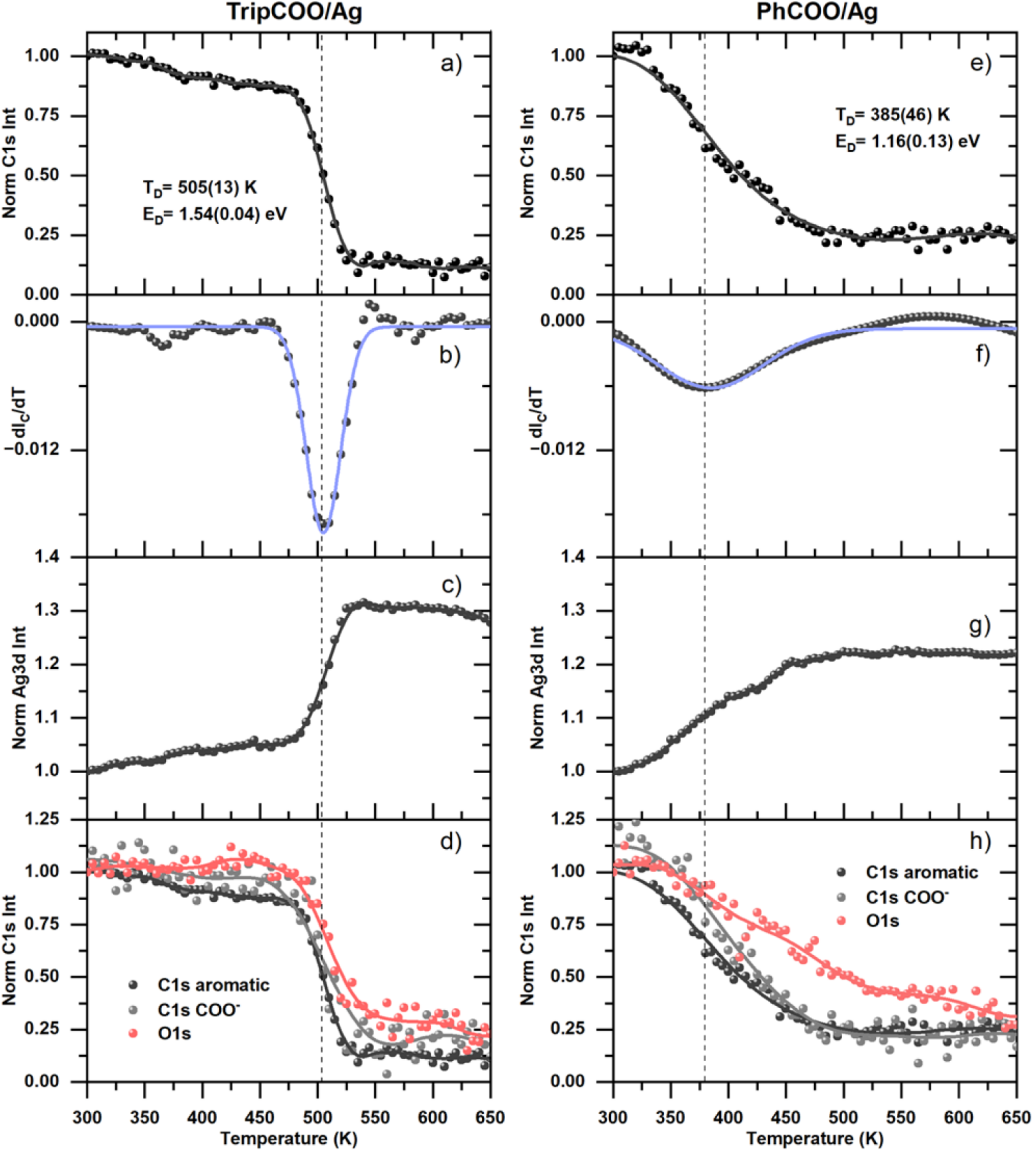
TP-XPS data for TripCOO/Ag (a-d) and PhCOO/Ag (e-h). (a,e): Temperature evolution of the C 1s intensity associated with the triptycene (a) and phenyl (e) backbones. (b,f) Smoothed derivatives of the curves shown in (a) and (e), respectively, used for determining *T*_D_ by fitting Gaussian peaks (blue lines) to the main minima. (c,g) Temperature evolution of the Ag 3d intensity associated with the substrate. (d,h) Temperature evolution of the C 1s and O 1s intensities associated with the aromatic backbone (black line) and carboxylate (gray and red lines). The vertical dashed lines indicate the *T*_D_ values, defined by the minima in (b) and (f), and serve as guides to the eye.

Analogous TP-XPS data for PhCOO/Ag are shown in the right panels of Figure 3. The desorption temperature of ∼385 K (Figure 3f), estimated for PhCOO/Ag as the minimum of a broad desorption peak (reflecting higher structural inhomogeneity of this monolayer consistent with the above NEXAFS data analysis), is ∼110 K lower compared to TripCOO/Ag, demonstrating a fundamental difference in the thermal stability of both monolayers. This difference is also visible when comparing the signals from the anchoring carboxylate groups. Whereas the C 1s signals originating from the carboxylate group and phenyl backbone in PhCOO/Ag show similar behavior (Figure 3h), the evolution of the O 1s signal from the carboxylate group occurs more slowly with increasing temperature than the C 1s signals. This means that some oxygen atoms are left after the desorption of the monolayer, which can be roughly estimated (considering the signal attenuation for the complete monolayer) as ∼30%. This observation shows that the relative thermal stability of the O–Ag and C–O bonds in PhCOO/Ag is different from TripCOO/Ag, indicating that the lower thermal stability of the former system can be caused by the reduced stability of the C–O bond in the anchoring groups.

To directly compare the thermal stability of PhCOO/Ag and TripCOO/Ag with that of the archetypal aliphatic SAMs on the same substrate, we performed reference TP-XPS experiments for HDT/Ag and heptadecenoic acid/Ag (CH_3_-(CH_2_)_16_-COO/Ag, HDCOO/Ag). The data are presented in Figure S4. Importantly, the desorption temperatures for HDT/Ag (∼427 K) and HDCOO/Ag (∼403 K) are both much lower compared to TripCOO/Ag (∼505 K), while at the same time, they are higher than the desorption temperature for PhCOO/Ag (∼385 K). This comparison again underlines a fundamental difference in the thermal stabilities between PhCOO/Ag and TripCOO/Ag.

The recorded desorption temperatures generally depend on the heating procedure; therefore, to compare our results with the literature data, we calculated the respective desorption energies (*E*_D_) using the Redhead equation^62^ (see the Supporting Information for details). The calculated values are summarized in Table 1. First of all, the desorption energy obtained here for HDT/Ag using TP-XPS (∼1.29 ± 0.04 eV) is consistent with previous estimates of this parameter (∼1.4 eV) obtained by using completely different experimental approaches, such as temperature-programmed desorption (TPD)^63^ and temperature-programmed secondary ion mass spectrometry (TP-SIMS).^29^ Most importantly, however, the calculated desorption energies for TripCOO/Ag (∼1.51 ± 0.05 eV) and PhCOO/Ag (∼1.16 ± 0.13 eV) can now be compared with longer aromatic SAMs formed on the Ag substrate using carboxylate anchoring group, such as those based on naphthalene (*E*_D_ ∼ 1.52 eV)^29^ and anthracene (*E*_D_ ∼ 1.64 eV),^17^ for which the respective desorption energies were estimated using the TP-SIMS technique. The comparison with the longer acenes emphasizes the limited thermal stability of the shortest aromatic monodentate SAMs, featuring a single phenyl ring as the backbone, which precludes real-life applications of such monolayers in organic electronics and photovoltaics. In contrast, the triptycene-based backbone clearly solves this problem by providing better SAM quality and tripodal adsorption geometry and elevates the thermal stability of such thinnest aromatic SAMs to the same level as that of analogous, few-times thicker aromatic monodentate monolayers.

### 2.3 Chemical Stability Analysis

To assess the chemical stability of TripCOO/Ag and PhCOO/Ag, we monitored their exchange by alkanethiols (HDT), which are capable of forming SAMs on Ag, as a result of incubating these monolayers in a solution containing the HDT molecules. The exchange process was monitored using infrared reflection−absorption spectroscopy (IRRAS) and XPS, and the results are summarized in Figure 4. The IRRAS data obtained for TripCOO/Ag were collected from the same sample after its incubation in a 1 mM ethanolic solution of HDT for a time ranging from 10 min to 7 days (Figure 4a). To trace the exchange process by IRRAS, we monitored two spectral ranges with the most intense bands, which are characteristic of TripCOO and HDT. Specifically, for TripCOO/Ag, the band corresponding to the symmetric carboxylate stretching (*v*_s_ COO^–^) at ∼1401 cm^–1^ was analyzed, which, complementary to XPS, is a fingerprint of the bidentate bonding of the molecules to the Ag substrate.^15,16,64,65^ For HDT, the symmetric and asymmetric C–H stretching bands of the aliphatic chain at ∼2964 cm^–1^ (*v*_a_ CH_3_), ∼2935 cm^–1^ (*v*_s_ CH_3_), ∼2919 cm^–1^ (*v*_a_ CH_2_), ∼2878 cm^–1^ (*v*_s_ CH_3_), and ∼2851 cm^–1^ (*v*_s_ CH_2_)^66^ were monitored (see the top spectrum in Figure 4a for the band assignment). Assuming that the surface coverage of TripCOO (0 ≤ θ_TripCOO_ ≤ 1) is proportional to the intensity of the *v*_s_ COO^–^ band, we can notice only a small drop in this parameter during 7 days of incubation. The coverage of the exchanged HDT can then be defined as θ_HDT_ = 1 – θ_TripCOO_ and its increase over the incubation time saturates at ∼10–20% after the first 10 min (Figure 4c). This result shows that TripCOO/Ag is strongly resistant to HDT exchange. Following previous studies of the chemical stability of SAMs performed on the basis of molecular exchange,^28,67^ it can be assumed that, in the present case, the exchange process is limited to some defects and domain borders in the monolayer and does not progress beyond the initial stage involving these defects. This scenario is also confirmed by the analysis of the HDT signal, which exhibits drastically different intensities of the C–H stretching bands compared with HDT/Ag (Figure 4a). These changes include, in particular, the dominating intensity of asymmetric (∼2919 cm^–1^) and symmetric (∼2851 cm^–1^) C–H stretching modes of the CH_2_ units, which have related TDM vectors perpendicular to the axis of the aliphatic chains.^66^ Considering surface selection rules (SSR) for IRRAS,^68^ the increased intensity of these bands results from a significantly more tilted orientation of the aliphatic chains, and thus, a much larger normal (to the metal surface) component of the related TDMs. The much more tilted orientation of the aliphatic chains is consistent with the adsorption of the HDT molecules only at the defect sides, which precludes the formation of an upright-oriented structure characteristic of the well-defined structure of HDT on Ag. The change in the HDT coverage as a function of incubation time for the TripCOO/Ag sample is shown in Figure 4c and can be fitted (gray dashed line) by the first-order Langmuir adsorption function^69,70^ (considering that in this case, adsorption is saturated at a specific coverage) in the form:

1 $$\theta_{HDT}\left(t\right)=\theta_{SAT}\left(1-e^{-ckt}\right)$$

**Figure 4:**
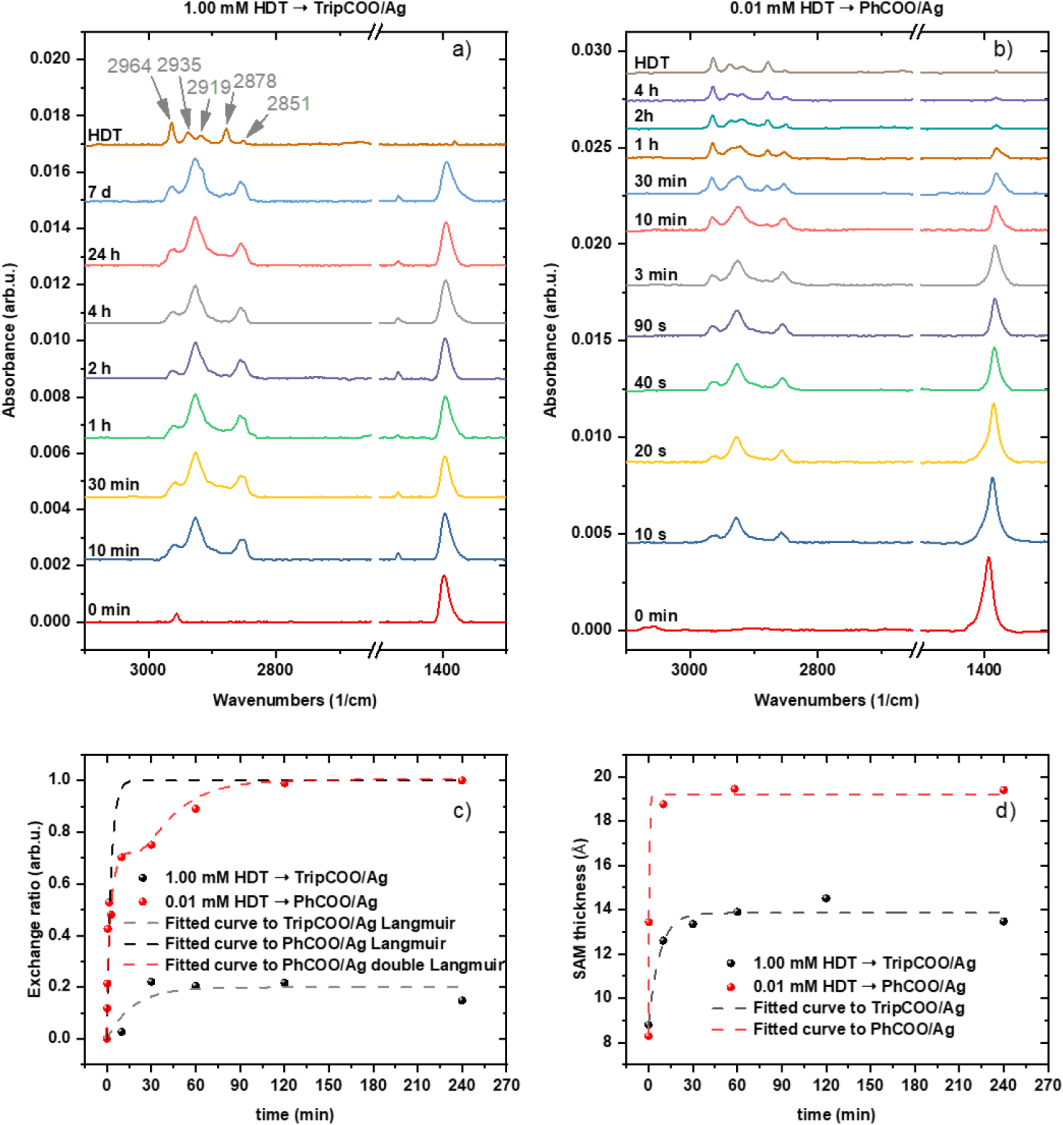
Summary of IRRAS and XPS data for the exchange of TripCOO/Ag and PhCOO/Ag by HDT. (a) IRRAS spectra of TripCOO/Ag recorded after its incubation into a 1 mM ethanolic solution of HDT; the incubation time is marked on the spectra. (b) IRRAS spectra of PhCOO/Ag recorded after its incubation into a 0.01 mM ethanolic solution of HDT; the incubation time is marked on the spectra. (c) The surface coverage of HDT as a function of the incubation time for TripCOO/Ag and PhCOO/Ag calculated from the respective IRRAS data; the fitted lines correspond to the first-order adsorption process. (d) The thicknesses of TripCOO/Ag and PhCOO/Ag calculated from the XPS data as functions of the incubation time.

where *c*, *k*, and θ_SAT_ are the concentration of adsorbing molecules in solution (in mol L^–1^), the rate constant of adsorption (in L mol^–1^ s^–1^), and the saturation coverage, respectively. Setting *c* to 10^–3^, we obtained the rate constant of ∼0.9 L mol^–1^ s^–1^.

The defect-limited exchange process of TripCOO/Ag by the HDT molecules, which saturates within the first ∼10 min, is also clearly visible in the evolution of the film thickness (estimated by XPS). This parameter changed from the value characteristic of TripCOO/Ag (∼8.8 Å) to ∼13.7 Å within the first 20–30 min of incubation and remained invariant afterward (Figure 4d). The latter value is well below the thickness characteristic of HDT/Ag (∼20.4 Å), reflecting, in accordance with the IRRAS data, the limited extent of the exchange reaction. The evolution of the TripCOO/Ag thickness can be fitted by the same type of mathematical function as (1); however, the rate constant for the exchange cannot be extracted since the thickness/coverage relation is not well-defined in this case.

The corresponding exchange process for PhCOO/Ag shows drastic differences. First of all, the exchange of the primary SAM by the HDT molecules is fully completed within just seconds using an HDT solution of the same concentration as that used for the experiments with TripCOO/Ag. Therefore, to monitor the exchange process in this case, the concentration of HDT was lowered by a factor of 100, down to 0.01 mM. The evolution of the IRRAS spectra of PhCOO/Ag during the exchange process under these conditions shows two main stages (Figure 4b). During the initial stage, which occurs within 3–10 min of incubation, the spectra in the HDT range resemble those recorded in the TripCOO/Ag case: they are dominated by the bands associated with asymmetric (∼2919 cm^–1^) and symmetric (∼2851 cm^–1^) C–H stretching modes of the CH_2_ units, indicating a strongly tilted orientation of the HDT chains. With the increase in incubation time above 30 min, the second stage of exchange takes place. The intensities of the above bands decrease, and the spectra, dominated by asymmetric (∼2964 cm^–1^) and symmetric C–H stretching modes of the terminal CH_3_ groups, gradually transform into those characteristic of HDT/Ag. The changes in the HDT surface coverage (θ_HDT_ = 1 = θ_PhCOO_) as a function of the incubation time can be monitored using the intensity of the carboxylate stretching band (∼1401 cm^–1^), but in contrast to TripCOO/Ag, the coverage evolution for PhCOO/Ag cannot be properly fitted by eq 1 in the whole range of the exchange process (see the dark gray dashed line in Figure 4c). However, a reasonable fitting is possible when separating the exchange process into two consecutive processes taking place in two independent time regimes (0–30 and 30–240 min), reflecting the two stages of the HDT structure during adsorption (as discussed above). These adsorption processes can be described as a sum of two Langmuir adsorption functions (with θ_SAT_ = 1) multiplied by the respective sigmoidal functions, which allow the separation of these two consecutive processes in time in the following form:

2 $$\theta_{HDT}\left(t\right)=A\left(t\right)\left(1-e^{-ck_{1}t}\right)+B\left(t\right)\left(1-e^{-ck_{2}t}\right)$$

$$A\left(t\right)={\left(1+e^{t-t_{1}/s_{1}}\right)}^{-1},\;B\left(t\right)={\left(1+e^{t_{2}-t/s_{2}}\right)}^{-1}$$

where *A*(*t*) and *B*(*t*) are decreasing and increasing sigmoidal functions, respectively, and *t*_1_, *t*_2_, *s*_1_, and *s*_2_ are respective parameters. The rate constants for both stages of adsorption obtained from this fitting procedure are distinctly different, being ∼588.0 L mol^–1^ s^–1^ (*k*_1_) and ∼72.0 Lmol^–1^ s^–1^ (*k*_2_) for the first and second stages, respectively. Most importantly, however, is the fact that both rate constants are several orders of magnitude higher than that of TripCOO/Ag (*k* ∼ 0.9 L mol^–1^ s^–1^). In general, we can conclude from these experiments and data analysis that PhCOO/Ag is unstable toward the exchange by alkanethiols, in contrast to TripCOO/Ag, which remains nearly fully resistant to this process.

## 3 Conclusions

In the present study, we compared the structure and thermal and chemical stability of the thinnest possible aromatic SAMs, assembled either in a monopodal or tripodal fashion on silver substrates. These one-phenyl-ring-thick monolayers feature either a phenyl or triptycene backbone in combination with carboxylate anchoring group(s). Spectroscopic analysis by XPS and NEXAFS spectroscopy confirmed, in both cases, the formation of well-defined, upright-oriented monolayers, with all available anchoring groups forming bidentate bonds to the substrate. These systems thus enabled a rational analysis of the impact of the tripodal versus monopodal bonding configuration on the stability of such analogous application-relevant SAMs. The thermal stability analysis, performed by TP-XPS, revealed a higher (by ∼0.4 eV) desorption energy for the tripodal system (TripCOO/Ag) compared to that of the monopodal one (PhCOO/Ag). For TripCOO/Ag, this energy even exceeded that of the thicker, long-chain alkanethiolates (by ∼0.2 eV). The chemical stability analysis, performed by IRRAS and XPS, demonstrated the fundamentally different stabilities of both monolayers toward exchange with alkanethiols in solution, with nearly perfect resistivity in the case of TripCOO/Ag and very low resistivity for PhCOO/Ag.

The above results clearly show that the triptycene framework provides a simple and rather unique vehicle for the functionalization of metal electrodes in devices by aromatic monolayers, which are at the same time ultrathin and highly stable, both thermally and chemically. Such a design concept is particularly appealing for organic electronics and photovoltaics, where the thickness of functional aromatic SAM, reduced to its physical limit, maximizes the charge transport over the metal–organic interface, and the high thermal/chemical stability warrants durability during the process of assembly and later operation of the respective device. Note that the triptycene framework can be flexibly decorated with functional tail groups, allowing the adjustment of energy level alignment and OSC morphology in devices.^24^

## 4 Methods

### 4.1 General Comments

The basic SAM characterization was performed by XPS and NEXAFS spectroscopy. The thermal stability analysis was conducted using TP-XPS. The chemical stability of the SAMs was monitored by IRRAS and XPS.

### 4.2 Sample Preparation

Two types of Ag(111) substrates were prepared by evaporation of Ag (99.99%, Testbourne Ltd., USA) film on either a silicon wafer (Si-Mat, Germany) or mica (V1 grade, Ted Pella, USA). The Ag/Si substrates were prepared by evaporation (RT, rate ∼ 0.01 nm/s) of ∼5 nm of an adhesive layer of chromium, followed by ∼100 nm of Ag film (RT, rate ∼ 0.1 nm/s), and were used for spectroscopic characterization and exchange experiments. The Ag/mica substrates were used for thermal stability studies. They were prepared by evaporation of ∼100 nm Ag film (∼360 °C, rate ∼ 0.1 nm/s) on freshly cleaved mica substrates, which were annealed at ∼360 °C for ∼24 h before the evaporation to remove water and surface contamination. The synthesis of 1,8,13-tricarboxytriptycene (TripCOOH) compound was conducted following the procedure described in our former study.^19^ Benzoic acid (PhCOOH) was purchased from the company (>99.5%, Sigma-Aldrich) and used as received. For the formation of SAMs, freshly prepared Ag(111) substrates were incubated in absolute ethanol (99.8% purity, Stanlab, purged for the last 30 min in N_2_) solution of either TripCOOH or PhCOOH for a specific time and at a defined temperature, and subsequently washed with absolute ethanol, followed by drying in a nitrogen stream. The TripCOO/Ag monolayers were obtained by 20 h immersion of Ag substrates into a 0.1 mM solution of TripCOOH at 65 °C.^19^ For PhCOO/Ag, 10 min incubation into a 0.5 mM solution of PhCOOH at RT was applied.^18^

As a reference for the spectroscopic experiments and thermal stability analysis, 1-hexadecanethiol (HDT, 97% Alfa Aesar) SAMs on Ag were prepared by 20 h of incubation of the Ag substrates in a 1.0 mM solution of HDT at room temperature (RT). As another reference for the thermal stability analysis, heptadecanoic acid (HDCOOH, ≥98% purity, Sigma-Aldrich) sample was prepared by 5 min of incubation of the Ag substrate in a 1.0 mM solution of HDCOOH at RT. For the IRRAS background correction, a perdeuterated hexadecanethiolate (PHDT) sample was used. The respective SAM was prepared by 20 h of immersion of the Ag substrate in a 1.0 mM solution of PHDT at RT.

### 4.3 XPS Measurements

The experiments were conducted by a ThermoFisher Scientific ESCALAB QXi Microprobe workstation using a monochromatized Al Kα X-ray source (*E* = 1486.6 eV) and a 650 μm spot size. All measurements were performed under ultrahigh vacuum (UHV) conditions, with a base pressure below 5 × 10^–10^ mbar. The spectra were acquired in the normal emission geometry with a 0.1 eV energy step, using the Ag 3d_5/2_ peak (368.2 eV)^71^ for the calibration of the BE scale. The data were collected and processed using Thermo Scientific Avantage software, and individual peaks were fitted with pseudo-Voigt profiles.

For the thermal stability measurements by TP-XPS, the same system was used following a recently published procedure.^72^ In short, the sample was heated at a rate of ∼3.6 K/min, and the XPS measurements were conducted with a 5 K step. The time of a measurement at a given temperature was only ∼27 s (to minimize possible temperature variation).^73^ Individual spectra were collected at different sample spots taken from the matrix of points evenly distributed over an area of ∼6 mm^2^ (to limit the potential irradiation-induced damage at elevated temperatures). To verify the sample homogeneity within this area, the substrate signal in the points of the matrix used for the thermal analysis was measured at RT, before the TP-XPS analysis, indicating excellent sample homogeneity.

XPS was also used for the chemical stability experiments, providing the effective thickness values for the TripCOO and PhCOO SAMs exposed to the HDT substituent for specific times.

### 4.4 NEXAFS Spectroscopy Measurements

The spectra were collected at the C K-edge using linearly polarized light (a polarization factor of ∼90%) at the HE-SGM beamline (bending magnet) of the synchrotron radiation facility BESSY II. The data were acquired in the partial electron yield detection mode with a retarding voltage of −150 V. The energy resolution was ∼0.3 eV. For the analysis of molecular orientation, the incident angle of the X-ray beam was varied in steps from 90° to 20° (with respect to the surface plane). The raw spectra were normalized to the spectrum of a freshly sputtered gold sample (to correct them for the energy dependence of the incident photon flux) and subsequently reduced to the standard form by setting the pre-edge intensity to zero and normalizing the far postedge region to the unity jump.^57,58^ As a reference for the photon energy scale, the π* resonance of highly oriented pyrolytic graphite (HOPG) at 285.38 eV was used.^74^

### 4.5 IRRAS Measurements

The IRRAS analysis was conducted using a Thermo Scientific Nicolet 6700 FT-IR spectrometer with a liquid-nitrogen-cooled MCT detector. Absorption spectra were collected with a resolution of 4 cm^–1^ using *p*-polarized light incident at a fixed angle of 80° with respect to the sample normal. All spectra are reported in absorbance units, *A* = −log *R*/*R*_0_, where *R* is the reflectivity of the SAM sample and *R*_0_ is the reflectivity of the reference sample (PHDT/Au). For the exchange experiments, consecutive spectra were taken for the same sample after different incubation times in ethanolic solutions of HDT. Before and after each measurement, the samples were washed in absolute ethanol and dried in a nitrogen stream.

## Supplementary Material



## Notes

Monolayer thickness analysis by XPS, average molecular tilt angle estimation by NEXAFS spectroscopy, and desorption energy estimation by TP-XPS (PDF)
