# Supplementary material for: Formation of Ultrathin and Highly Stable Aromatic Monolayers on Silver Surface—Three Legs Are Better Than One
Source: ACS Appl Mater Interfaces. 2026 Mar 3;18(10):15665–75. doi: 10.1021/acsami.5c19530 (PMC13298814; doi:10.1021/acsami.5c19530)
Supplement: Supplementary file 1 [file am5c19530_si_001.pdf]

Supporting Information

for

Formation of Ultrathin and Highly Stable Aromatic Monolayers on Silver Surface – Three Legs are Better than One.

Anna Rojek,[§] *Daria M. Cegiełka,*[§] *Mateusz Wróbel,*[§] *Magdalena Stępień,*[§] *Yoshiaki Shoji,*[#,‡] *Takanori Fukushima*[#,‡]* *Michael Zharnikov,*[†]* and *Piotr Cyganik*[§]*

[§] Jagiellonian University, Faculty of Physics, Astronomy and Applied Computer Science, Smoluchowski Institute of Physics, Łojasiewicza 11, 30-348 Kraków, Poland

[#] Laboratory for Chemistry and Life Science, Institute of Integrated Research, Institute of Science Tokyo, Yokohama 226-8501, Japan

[‡] Research Center for Autonomous Systems Materialogy (ASMat), Institute of Integrated Research, Institute of Science Tokyo, Yokohama 226-8501, Japan

[†] Angewandte Physikalische Chemie, Universität Heidelberg, Im Neuenheimer Feld 253, D-69120 Heidelberg, Germany

* Authors to whom correspondence should be addressed.

Piotr Cyganik (piotr.cyganik@uj.edu.pl),

Michael Zharnikov (Michael.Zharnikov@urz.uni-heidelberg.de)

Takanori Fukushima (fukushima@res.titech.ac.jp)

1. Monolayer thickness estimation by XPS

The ratio of the C 1s to Ag 3d peaks areas was used to estimate the thickness of the SAMs according to the equation

(1) $$\frac{I_{Ag3d}}{I_{C1s}} \exp\left(-\frac{d}{\lambda_{\mathrm{Ag3d}}}\right) = K\left(1 - \exp\left(-\frac{(d-z)}{\lambda_{\mathrm{C1s}}}\right)\right)$$

$$\lambda_{\mathrm{i}} = 0.3\ \mathrm{E}_{\mathrm{k_i}}^{0.64}$$

$$E_{k_i} = hv - \Phi_{\mathrm{XPS}} - E_{b_i}$$

where I_{C1s} and I_{Ag3d} are the total intensities of the C 1s and Ag 3d signals, respectively, K is the apparatus constant [unitless] which was estimated using the reference SAM with the well-known thickness, z is the thickness of the bonding group [Å] ($z_{(\mathrm{Ag\text{-}SC})}$=3.51 Å or $z_{(\mathrm{Ag\text{-}COO})}$=2.75 Å), d is the SAM thickness [Å], λ_{i} is the average electron mean free path [Å] for the photoelectrons emitted from the *i*-element, E_{k_i} is the kinetic energy of these electrons [eV], hv is the photon energy [eV] (1486.6 eV for Al Kα), Φ_{XPS} is the work function of the spectrometer [eV], and E_{b_i} is the binding energy of the corresponding signal. (1). As the reference, we used the well-characterized hexadecanethiolate (HDT) SAM on Ag; its thickness is 20.3 Å.

2. Average molecular axis tilt angle estimation by NEXAFS spectroscopy

2.1. PhCOO/Ag

For a vector-like orbital and a substrate with three-fold (or higher) symmetry, the intensity of an absorption resonance as a function of the X-ray incident angle, θ, is described by the formula[1]

(2) $$I(\theta) = A\left\{P\frac{1}{3}\left[1 + \frac{1}{2}(3\cos^2\theta - 1)(3\cos^2\alpha - 1)\right] + (1 - P)\frac{1}{2}\sin^2\alpha\right\},$$

where A is the orbital-specific constant, P is the X-ray polarization degree (0.9 in our experiments), and α is the angle between the direction of the vector orbital (the π_{Ph}* orbital in our case) and the substrate normal, which could be further recalculated to the average molecule tilt angle β assuming the value of molecules twist angle σ (see **Figure S1**).

Since the constant A value is unknown, we calculate the relative intensities, $I(\theta)/I(20°)$, $I(\theta)/I(30°)$, $I(\theta)/I(55°)$, and $I(\theta)/I(90°)$ of the π_{Ph}* resonance. The respective data were fitted in accordance with eq. 1 to calculate the α angle (see **Figure S2**). In the next step, the average over the α values derived from the $I(\theta)/I(20°)$, $I(\theta)/I(30°)$, $I(\theta)/I(55°)$, and $I(\theta)/I(90°)$ data fitting was calculated and used for the calculation of the average molecular tilt angle for the particular monolayer using eq. 2. A twist angle σ 32°, characteristic of bulk phenyl,[2] was assumed.

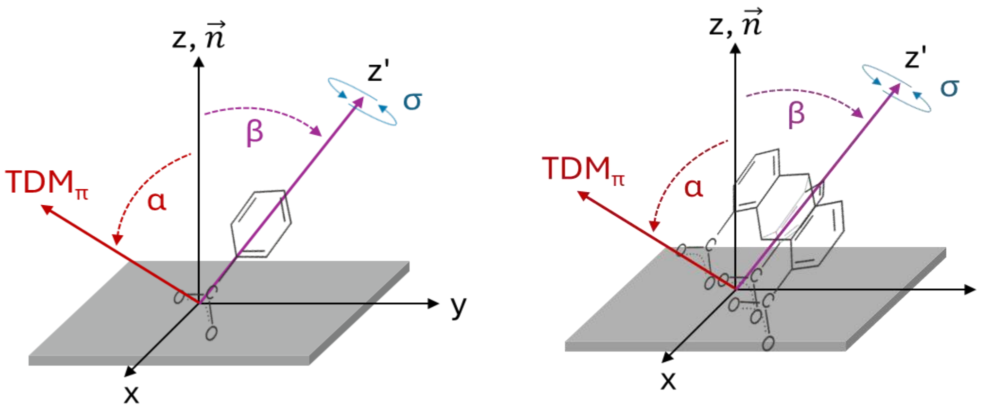

Figure S1. Scheme of the relevant angles for the analysis of the molecular orientation in PhCOO/Ag (left drawing) and TripCOO/Ag (right drawing), where z-axis is the metal substrate normal. The main molecule axis (z') is tilted against the surface normal (z-axis) by the angle β. The TDM vector is tilted against the surface normal by the angle α. The rotation of the molecule about the main axis is described by the twist angle σ. This angle is considered as zero if the tilt plane is perpendicular to the plane of the phenyl ring.

2.2. TripCOO/Ag

For the SAMs based on triptycenes, the threefold symmetry of the molecule enables a simplified analysis. Here, each out of three aromatic rings yields independent NEXAFS signal of equal intensity with the TDMs rotated by 120° with respect to each other (see **Figure S1**). The resulting intensity can be described by the following equation[3]

(3) $I(\theta) = \frac{3B}{2} P(3\sin^2\beta - 1)\cos^2\theta + \frac{3B}{2}\left(1 - \frac{1}{2}\sin^2\beta\right)$

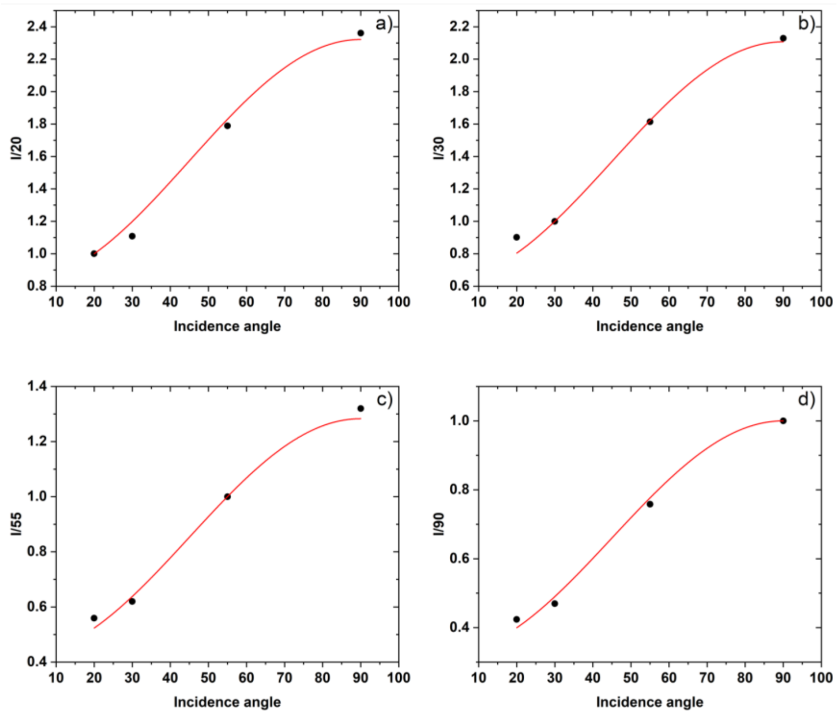

Figure S2. NEXAFS data for PhCOO/Ag analysis: (a) $I(\theta)/I(20°)$, (b) $I(\theta)/I(30°)$, (c) $I(\theta)/I(55°)$, and (d) $I(\theta)/I(90°)$ (see text for details).

where B and P are the scaling factor and the X-ray polarization factor, respectively. Plotting the relative resonance intensities versus $cos^2\theta$ yields a linear relationship (see **Figure S3**). It allows an easy determination of β using the equation[3]

$$(4) \quad \beta = \sin^{-1}\left(\sqrt{\frac{2m+2aP}{m+3aP}}\right)$$

where m and a are the slope and intercept of the linear fit, respectively. Thus, this method provides a direct determination of β in triptycene-based SAMs, overcoming the limitations associated with the assumption of the σ angle value. The average value of β from the $I(\theta)/I(20°)$, $I(\theta)/I(30°)$, $I(\theta)/I(55°)$, and $I(\theta)/I(90°)$ fittings was taken as the average molecular tilt angle reported in **Table 1**.

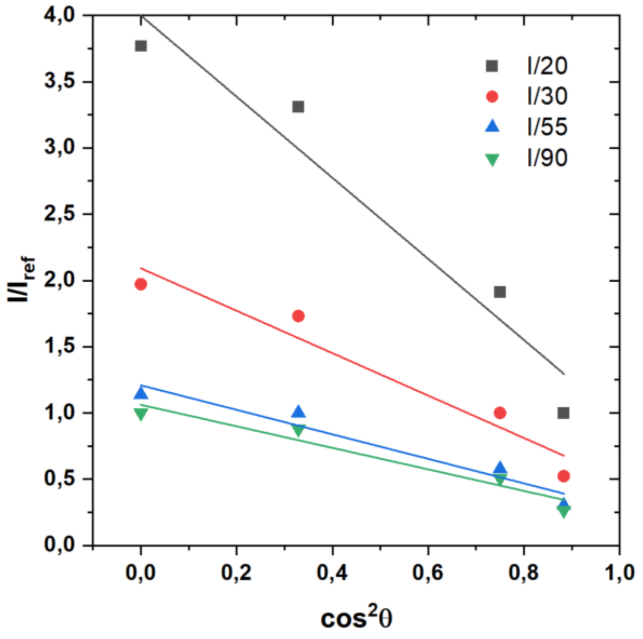

Figure S3. NEXAFS data analysis for TripCOO/Ag (see text for details).

3. TP-XPS data for HDT/Ag and HDCOO/Ag

4. Desorption energy estimation by TP-XPS

To analyze the thermal desorption process, the intensity of the main component of the C 1s spectra (Figure 2a), normalized to the value at the room temperature, was plotted versus temperature for each particular SAM. Subsequently, the plots were smoothed using FFT filter method and the first derivative of them was taken. Then, the desorption temperature T_D and the corresponding error (standard deviation) of this parameter were estimated by fitting Gaussian function to the minimum of the derivative curves. Finally, the desorption energy E_D (eV) and the corresponding error were calculated using the Redhead equation[4]

(5) $$E_D = T_D \left[\ln\left(\frac{\nu_T T_D}{s}\right) - 3.64\right]$$

where ν_T is the preexponential (frequency) factor (typically 10^{13} Hz for SAMs[5]) and s is the heating rate (~3.6 K/min).

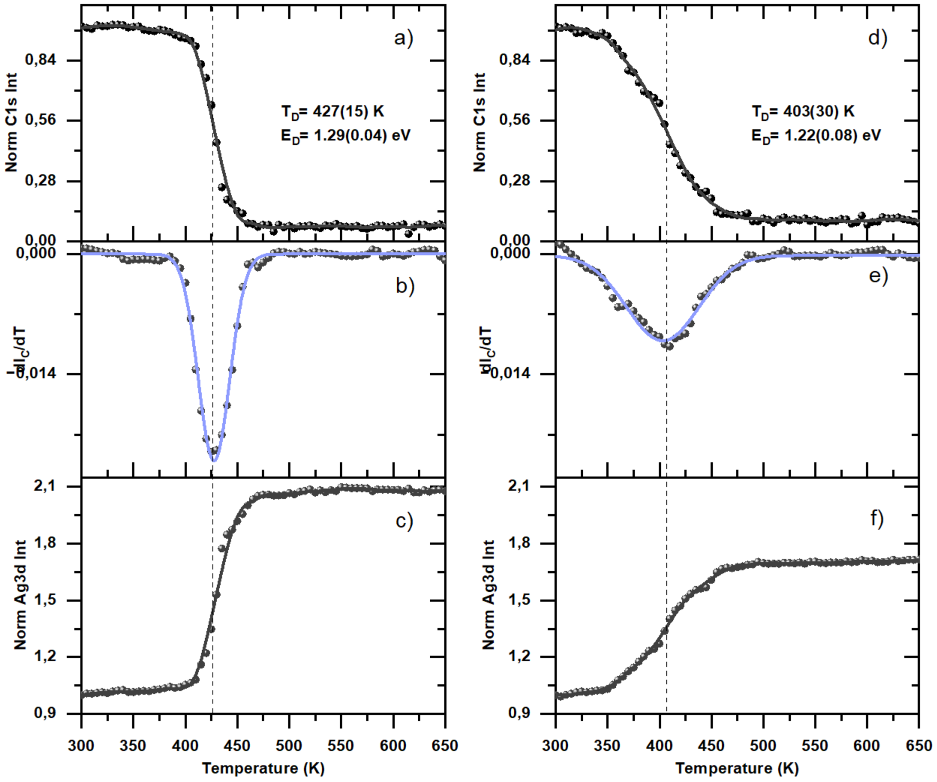

Figure S4. STP-XPS data for HDT/Ag (left panel) and HDCOO/Ag (right panel). In (a) and (d) temperature profile for the C 1s intensity associated with the aliphatic backbone. In (b) and (e) smoothed derivative of this profile used for determining the desorption temperature by fitting gaussian peak (blue line) to the main minima. In (c) and (f) temperature profile for the Ag 3d substrate intensity. In (g) profile for the C 1s intensity associated with the aromatic backbone (black line) and carboxylate (red line). The vertical dashed line indicates the mean of the desorption temperature values (defined by the minimum in (b) and (e) profiles) and serves as a guide to the eye.

References

(1) J. Stöhr, NEXAFS Spectroscopy; Springer Series in Surface Sciences; Springer: Berlin, 2003.

(2) Zhang, C.; Das, S.; Sakurai, N.; Imaizumi, T.; Sanjayan, S.; Shoji, Y.; Fukushima, T.; Zharnikov, M. Phosphonic acid anchored tripodal molecular films on indium tin oxide. *PCCP* **2024**, *26*(15), 11360–11369.

(3) Liu, J.; Kind, M.; Schüpbach, B.; Käfer, D.; Winkler, S.; Zhang, W.; Terfort, A.; Wöll, C. Triptycene-Terminated Thiolate and Selenolate Monolayers on Au(111). *Beilstein Journal of Nanotechnology* **2017**, *8*, 892–905.

(4) Redhead, P. A. Thermal Desorption of Gases. *Vacuum* **1962**, *12*, 203–211.

(5) Lavrich, D. J.; Wetterer, S. M.; Bernasek, S. L.; Scoles, G. Physisorption and Chemisorption of Alkanethiols and Alkyl Sulfides on Au(111). *J. Phys. Chem. B* **1998**, *102*(18), 3456–3465.
